# Medical Students’ Perceptions of Clinical and Research Training: An International Needs Assessment of 26 Countries

**DOI:** 10.21106/ijtmrph.454

**Published:** 2023-09-23

**Authors:** Sergio M. Navarro, Kelsey Stewart, Katelyn M. Tessier, Aemon Berhane, Susana Prestinary Alvarado, Tavonga Tafirei, Hodan Abdi, Evan J. Keil, Todd Tuttle, Jennifer Rickard

**Affiliations:** 1 University of Minnesota, Department of Surgery, Minneapolis, MN, US; 2 University of Minnesota, Department of Obstetrics and Gynecology, Minneapolis, MN, US; 3 Masonic Cancer Center, Biostatistics Core, University of Minnesota, Minneapolis, MN, US; 4 Medicine at College of Health Sciences, Addis Ababa University, Addis Ababa, Ethiopia; 5 Universidad de Ciencias Medicas, San Jose, Costa Rica; 6 National University of Science and Technology, Zimbabwe, Bulawayo, Zimbabwe; 7 Brigham and Women’s Hospital, Department of Surgery, Boston, MA; 8 University of Michigan, Department of Obstetrics, Ann Arbor, MI, US; 9 University of Rwanda College of Medicine and Health Sciences, Kigali, Rwanda

**Keywords:** Medical Student, Medical School, Survey, Research, International, Global Surgery

## Abstract

**Objective:**

Despite calls to incorporate research training into medical school curriculum, minimal research has been conducted to elucidate trends in research knowledge, opportunities, and involvement globally. This study aims to: (1) assess medical students’ perceptions of the level of training they received on research based on their medical school training, and (2) evaluate the obstacles related to conducting research as part of medical students’ training.

**Methods:**

A 94-question, bilingual survey designed by a small focus group of individuals from medical schools across the globe and administered to medical students from different parts of the world, distributed via social media networks (Twitter, Now X, Facebook) and email distributions via international partnerships from November 1 to December 31, 2020. The survey collected demographic information including age, gender, medical institution and country, degree, year in training, clinical rotations completed, plans for specialization, and additional graduate degrees completed. Statistical analysis included a summary of survey participant characteristics, and a comparison between regions, with a variety of comparison and logistic regression models used.

**Results:**

A total of 318 medical students from 26 countries successfully completed the survey. Respondents were majority female (60.1%), from Latin America (LA) (53.1%), North America (NA) (28.6%), and Other world regions (Other) (18.2%). Students felt research was an important component of medical training (87.7%), although many reported lacking research support from their institution (47.5%). There were several reported barriers to research, including lack of research opportunities (69.4%), lack of mentors (56.6%), lack of formal training (54.6%), and barriers due to the coronavirus disease 2019 (COVID-19) pandemic (49.3%). Less frequent were barriers related to financial resources (41.6%), physical resources (computer or internet access) (18%), and English language ability (6.9%). Students from Latin America and Other were more likely to report a desire to pursue research later in their medical careers compared with students from North America.

**Conclusions and Implications for Translation:**

Despite significant interest in research, medical students globally report a lack of formal research training, opportunities, and several barriers to conducting research, including the COVID-19 pandemic. The study highlights the need for student research training internationally and the role of further regional-specific and institutional-specific evaluation of research training needs.

## 1. Introduction

The current landscape of medical students’ knowledge, resources, and barriers to performing research across the globe is still being understood. Early involvement in medical research can be foundational to the career of a physician in training and the benefits of such involvement extend in all directions.^1^ For medical trainees, their involvement is associated with more informed career choices, improved scientific productivity both in the long and short term, increased knowledge of research, and increased interest in research.^2,3^ For attending physicians, their research productivity is often increased with the involvement of medical students.^4^ The involvement of medical students may also prove to be critical for the future of research in certain areas of the globe.^5^

Given this importance, the implementation of graduation theses (adopted in countries such as Germany), intercalated degrees, and other research-related graduation requirements have been instituted or proposed to increase medical trainees’ involvement in research.^6–8^ Further evaluation is underway to evaluate the benefit of medical student education in research efforts globally.^9^ These recommendations and requirements are often well received by students given the positive impact on their futures and general interest in conducting research.^10^ There appear to be near equal desires among medical students to conduct research both within high and low-income countries.^11^

However, even with the influx of calls for medical education reform and the positive influence of research at the medical student level, minimal research has been conducted to elucidate trends in research knowledge, opportunities, and involvement globally. Over 50% of published studies related to medical student interests and involvement in research between 1985 and 2018 were based in the United States and the United Kingdom.^12^ However, research conducted to evaluate barriers to research has been primarily based outside of the United States.^2,13^ Unawareness of home institution research, knowledge deficits, lack of formal training, and lack of funding were common barriers.^2,13^ Studies primarily evaluate barriers at a single institution, infrequently at multiple institutions, and rarely across international borders.

The aim of this study was to address the knowledge gap of the barriers, exposure, and experience with research at the medical student level and provide more information on regional differences globally affecting medical students wishing to participate in research.

## 2. Methods

### 2.1. Participants

Any medical student enrolled in a learning institution, including national and international sites, was deemed eligible for inclusion. Co-author organizations other than the University of Minnesota included the Universidad de Ciencias Médicas de Costa Rica, Addis Ababa University, Ethiopia; the University of Rwanda, and the University of Science and Technology in Zimbabwe.

Ethical review was completed prior to disseminating the survey by the University of Minnesota Institutional Review Board. The study (STUDY00010789) was deemed non- human research and was exempt from further review.

### 2.2. Survey Design

The survey was designed based on a collective review by a small focus group of individuals from medical schools across the globe, with representatives from Costa Rica, Ethiopia, Zimbabwe, and the United States. The survey collected demographic information including age, gender, medical institution and country, degree, year in training, clinical rotations completed, plans for specialization, and additional graduate degrees completed. Participants answered questions pertaining to six key topics - (1) clinical experience, (2) research perceptions, (3) research experience, (4) research resource availability, (5) research skills, and (6) barriers to research during medical school. Questions primarily consisted of ranking questions (on a scale from 1–5), yes or no questions, multiple response questions, and short answer questions. A total of 94 questions were included in the survey, including 9 demographic questions. The study was conducted via an online survey hosted on Google Forms (Google, Mountain View, United States).

### 2.3. Survey Dissemination

The survey was distributed to medical students through social media networks, namely Twitter (Now X) and institutional Facebook social networks, and specific global surgery network electronic mailing lists, namely Global Student Survey Alliance (GSSA) and Incision, from November 1 - December 31, 2020. None of the survey participants were individually contacted.

### 2.4. Data Analysis

All partially completed responses were considered for inclusion in the analysis after the exclusion of duplicate responses. Survey responses were summarized for all subjects using descriptive statistics. Analysis was performed comparing subjects by key region, namely North America (NA), Latin America (LA), and Other Regions (Other). A full map of region classification by country is found in footnote A of Table 1. Regional breakdown analysis was conducted comparing responses from the NA region, LA region, and Other regions. To investigate the association between region and continuous participant characteristics, analysis of variance (ANOVA) was used; Chi-square or Fisher’s exact tests were used for categorical characteristics. To investigate the effect of region on interests in medical student participation in research and barriers to participation in research, logistic regression models were used. Odds ratios (OR) and 95% confidence intervals (CI) comparing LA and “Other” to NA were obtained. Fisher’s exact tests were used for some variables due to model fit. All regression models were adjusted for age, gender, and the percentage of years of training completed. All reported p-values are two-sided and a significance level of 0.05 was used unless otherwise stated. Statistical analyses were performed using R (version 3.6.1, R Core Team) and SAS (version 9.4, SAS Institute Inc., Cary, North Carolina).

## 3. Results

### 3.1. Survey Respondent Characteristics

A total of 318 medical students from 26 countries and 44 medical schools completed the survey. Respondent characteristics can be found in Table 1. Respondents were majority female (190, 60.3%) and had a mean age of 23. A majority were from LA (169, 53.1%), followed by NA (91, 28.6%), and Other regions (58, 18.2%). Students had a median of 2 and 4 months of research and clinical experience, respectively. Nearly 9% (n=27) of students had an additional graduate-level degree.

### 3.2. Research Interests and Experience

Most respondents were interested in conducting research as a medical student (277, 87.1%) and as a future physician (187, 58.8%). Overwhelmingly, students felt that research is an important component of medical training (277, 87.7%). However, many students were not required to participate in research to graduate from training (183, 58.1%), and had not participated in research (164, 52.4%). Many students reported a lack of research support from their institution (149, 47.5%).

Notable differences were found by region among medical students’ interests and experience in research (Table 2). Overall, LA (120, 71.0%; adjusted OR (aOR): 2.68; 95% CI: 1.35, 5.31) and Other (52, 89.7%; aOR: 7.96; 95% CI: 2.85, 22.28) students reported a higher interest in research later in their medical training compared to NA students (53, 58.2%, p<0.001). This compares similarly to views for later in their careers with LA (103, 60.9%; aOR: 3.80; 95% CI: 1.96, 7.36) and Other (48, 82.8%; aOR: 9.87; 95% CI: 4.02, 24.22) when compared to their NA counterparts (36, 39.6%, p<0.001). NA students (61, 70.1%) were more likely to have participated in research compared to those in LA (35, 20.7%; aOR: 0.10; 95% CI: 0.05, 0.20) and Other (10, 17.5%; aOR: 0.10; 95% CI: 0.04, 0.24; p<0.001). Additionally, in LA (39, 23.8%) and Other (20, 35.1%), medical students were more likely to report a motivating factor of research as interest in being a physician-scientist compared to NA students (3, 3.4%, p<0.001). NA students (69, 77.5%) were more likely to report competitiveness for matching to specialty training after medical school as a motivating factor compared to LA (95, 57.9%; aOR: 0.44; 95% CI: 0.23, 0.83) and Other students (30, 52.6%; aOR: 0.37; 95% CI: 0.17, 0.79; p=0.027).

### 3.3. Barriers to Research

Reported barriers to research included lack of research project opportunities (211, 69.4%), lack of mentors (172, 56.6%), lack of formal training (166, 54.6%), and barriers due to the COVID-19 pandemic (150, 49.3%). Less frequent were barriers related to financial resources for publication/presentation (124, 40.8%), physical resources (computer or internet access) (16, 5.3%), and English language ability (21, 6.9%).

Notable differences were found by region-identifying barriers to research (Table 3). Barriers to research include lack of research mentors (p=0.005), lack of available research project opportunities (p=0.028), lack of physical resources (p=0.029), lack of research resources (p<0.001), lack of access to medical research articles (p<0.001), and lack of financial resources for publication and attendance for presenting presentations (p<0.001) were more frequent in the Other region compared to LA and NA. Additionally, an overall lack of “research focus” culture at their institution was lower compared to LA and Other regions (p<0.001). No differences were found by region in terms of time for research or willingness to participate in research with barriers removed.

## 4. Discussion

Overall, there is a broad interest in research among medical students globally. Early involvement in medical research may be foundational to the career of a physician in training as well as beneficial to the medical trainee in informing career choices and knowledge and interest in research.^2,3,14^ The involvement of medical students has been thought to help address key global medical research questions.^5,15^

Gaps remain in understanding trends in medical student-based research knowledge, opportunities, and involvement globally, with more than 50% of research over the past three decades based in the United States and the United Kingdom.^12^ Research on barriers to medical student-led research has found home institution research, knowledge deficits, lack of formal training, and lack of funding to be common barriers.^2,13,16^ This study sought to build upon existing work to further evaluate barriers and opportunities to medical student research participation at an international and global level.

NA students have more exposure to research, but less long-term interest in research as a career compared with students from LA and Other regions. One key finding of our research based on comparison by research found a statistically significant difference in interest and motivation to participate in research by region, namely there were statistically significant differences in medical students expressing conducting research as part of their physician career, with Sub-Saharan African students much more likely to express research interest later in their career. NA students were much more likely to report that their medical student colleagues were participating in research as a medical student. Furthermore, overall interest in being a physician-scientist was higher in Other and LA compared to medical students in NA.

There are several potential reasons for this. Early exposure to research by NA students may allow them to better inform their career aspirations. Opportunities for career growth and lifestyle may differ for academics in NA compared with LA and Other regions. For example, in the US, academics compared with private practice is often associated with lower pay. Whereas in other regions, research experience may open opportunities for broader career choices. Careers in public health or employment by NGOs may be more lucrative in certain countries/regions compared with strictly clinical work.

Respondents identified a lack of research training and education. Some medical schools around the world have instituted trainee involvement with research as part of their graduation requirement criteria in addition to the development of graduation theses to increase medical trainees’ involvement in research with positive feedback from trainees on a global level.^6–8,10,12–13,17–20^ However, this is not a uniform requirement. Certain organizations, including the Young British Journal of Surgery (BJS) and American College of Surgeons (ACS), provide free, available short courses on research as well as examples of how to develop a research protocol, which may help aspiring future physician-scientist researchers.

Research experience, opportunities, and mentorship are inadequate with the greatest deficits noted in LA and Other countries, compared with NA students. Education and coursework are helpful, but by itself, may be insufficient to develop requisite skills and experience in research. Ultimately, like clinical skills, students benefit from real-world experience to master research skills. This often includes an opportunity to work on a research project, as well as having the mentorship and guidance to be taught during the process. A database outlining research project opportunities may help medical students find potential projects and connect with collaborators. The GSSA has developed a network, along with supporting a resource database of faculty and residents with projects, available to any medical student who registers.^17^ Such a solution may be broadened from global surgery to other global topics and key issues. Mentorship opportunities to medical students for conducting research were found across regions to be a limiting factor. Additional institutional programs and resources dedicated to assigning each medical student a mentor may prove helpful in addressing this.

Research resources, including financial and material resources, remain a challenge. Not only do students need material resources to complete a research study, but financial resources for publication and presentation at conferences. Financial resources for presentation and publication were cited by students in LA and Other compared to students in NA, indicating that a program supporting research travel, cost of attendance, and presenting research may be beneficial to global medical students. Virtual conferences may help provide opportunities for students globally to present but may limit the value of in-person networking at conferences.

Regional analysis found that across regions, students reported being affected by COVID-19, with no difference between the regions found. This may indicate that a better curriculum or program able to meet the constraints of COVID-19 may be helpful for helping promote research participation by medical students regardless of location, whether one featuring an online curriculum or one taught locally based on a standardized global curriculum. Furthermore, programs supporting research initiatives that span across training environments, by connecting training at the medical school to residency training and junior faculty level career level may prove beneficial.

While this study was a global study, it may not be a fully comprehensive representative population of the broader array of global medical students, limited by the overall size of survey respondents and geographical distribution. Europe, Southeast Asia, the Middle East, and East Asia were regions with fewer responding medical students than North America, Latin America, and Sub-Saharan Africa, and may not be fully represented in this study. As this survey was conducted in the English and Spanish languages, it may have biased results to more English-language-speaking populations. This may have skewed participation towards those who are primary English or Spanish language speakers. Institutional variation may have skewed results in the survey, as students may have been from certain institutions and do not necessarily reflect all institutions within a given country. Furthermore, institutions may offer specific tracks in medical school curricula which may influence perspectives and introduce bias in respondents. Other limitations may include self-selection bias for those who filled out the survey being more interested in research as medical students compared to those who did not fill out the survey as well as bias due to varying involvement in social media where the survey was distributed and variable access to email, due to the use of both social media platforms and electronic mailing lists to distribute the research survey. We have not conducted additional rounds of review with survey participants to review results and available data collected, nor included survey participant information in this study.

Future research and work should be focused on exploring regional differences at an institutional level, with plans for a needs assessment study at partner institutions internationally in Latin America, Sub-Saharan Africa, and North America, to develop a potential pilot program for addressing barriers to medical student-led research work. This will address selection bias among participants in the current survey. Further surveys could be regionally specific as well as country-specific on the needs of medical students in those regions and countries. Future research will focus on developing surveys conducted in the primary language of participants as well as conducting additional rounds of review with participants. The publication of contact information of survey participants will be considered in future studies to provide a more complete online scientific survey list with detailed contact information to the community.

## 5. Conclusion and Implications for Translation

Despite significant interest in research, medical students globally report a lack of formal research training, opportunities, and several barriers to conducting research, including the COVID-19 pandemic. This survey demonstrates a need for student research training internationally.

## Figures and Tables

**Table 1:** Survey participant characteristics and demographic breakdown

	All participants (N=318)	Latin America (N=169)	North America (N=91)	Other (N=58)	p-value^D^
Age					<0.001
Median (Range)	23.0 (17.0, 49.0)	22.0 (17.0, 49.0)	25.0 (18.0, 34.0)	23.0 (19.0, 32.0)	
Mean (SD)	23.6 (3.4)	22.7 (3.6)	25.5 (2.2)	23.4 (2.8)	
Gender, n (%)					0.033
Female	190 (60.3)	99 (59.3)	64 (70.3)	27 (47.4)	
Male	122 (38.7)	67 (40.1)	26 (28.6)	29 (50.9)	
Non-Binary	3 (1.0)	1 (0.6)	1 (1.1)	1 (1.8)	
Region, n (%)^A^		-	-	-	-
Latin America	169 (53.1)				
North America	91 (28.6)				
Sub-Saharan Africa and Other	58 (18.2)				
Medical institution, n (%)		-	-	-	-
Addis Ababa University	8 (2.5)				
National University of Sciences & Technology	9 (2.9)				
University of California Davis	7 (2.2)				
University of Medical Sciences Costa Rica	146 (46.3)				
University of Minnesota	75 (23.8)				
University of El Salvador	15 (4.8)				
University of Rwanda	10 (3.2)				
Other^B^	45 (14.3)				
Medical School Degree, n (%)					-
MBBS	42 (13.2)	19 (11.2)	0 (0.0)	23 (39.7)	
MbChB/Other*	44 (13.8)	33 (19.5)	0 (0.0)	11 (19.0)	
MD	232 (73.0)	117 (69.2)	91 (100.0)	24 (41.4)	
Percentage of years training^C^					0.070
Median (Range)	66.7 (16.7, 125.0)	66.7 (16.7, 116.7)	50.0 (25.0, 125.0)	66.7 (16.7, 100.0)	
Mean (SD)	60.4 (26.5)	59.2 (27.0)	58.0 (27.1)	67.5 (23.5)	
Years of clinical experience required for your medical degree, n (%)					<0.001
<1 year	6 (1.9)	3 (1.8)	2 (2.2)	1 (1.7)	
1–2 years	18 (5.7)	13 (7.9)	2 (2.2)	3 (5.2)	
2–3 years	106 (33.8)	17 (10.3)	73 (80.2)	16 (27.6)	
3–4 years	89 (28.3)	67 (40.6)	0 (0.0)	22 (37.9)	
≥4 years	95 (30.3)	65 (39.4)	14 (15.4)	16 (27.6)	
Years of clinical experience completed, n (%)					<0.001
<1 year	158 (50.6)	65 (39.4)	71 (78.0)	22 (39.3)	
1–2 years	60 (19.2)	32 (19.4)	13 (14.3)	15 (26.8)	
2–3 years	48 (15.4)	30 (18.2)	7 (7.7)	11 (19.6)	
3–4 years	28 (9.0)	22 (13.3)	0 (0.0)	6 (10.7)	
≥4 years	18 (5.8)	16 (9.7)	0 (0.0)	2 (3.6)	
Years of research training required for your medical degree, n (%)					<0.001
<1 year	212 (70.0)	87 (55.1)	86 (94.5)	39 (72.2)	
1–2 years	43 (14.2)	30 (19.0)	4 (4.4)	9 (16.7)	
2–3 years	21 (6.9)	18 (11.4)	1 (1.1)	2 (3.7)	
≥3 years	27 (8.9)	23 (14.6)	0 (0.0)	4 (7.4)	
Years of research training completed, n (%)					<0.001
<1 year	237 (78.5)	103 (66.0)	85 (94.4)	49 (87.5)	
1–2 years	41 (13.6)	32 (20.5)	4 (4.4)	5 (8.9)	
2–3 years	12 (4.0)	10 (6.4)	1 (1.1)	1 (1.8)	
≥3 years	12 (4.0)	11 (7.1)	0 (0.0)	1 (1.8)	

A Breakdown of Regions; Latin America: Costa Rica, Brazil, El Salvador; Peru, Barbados, Haiti, Grenada; North America: United States, Mexico; Other: Ethiopia, China, Zimbabwe, Sweden, Nigeria, Rwanda, Australia, Nepal, South Africa, Uganda, UK, Ghana, Malawi, Kenya, Ireland, Turkey, Libya, India

B Other medical institution includes Mekelle University, Jimma University, Zinzhou Medical University, St Paul’s Hospital Millenium Medical College, Federal University of Amazonas, Peruana Cayetano Heredia University, Karolinska Institute, University of Illinois Chicago, University of Lagos, Ross University, University of Notre Dame Australia, St George’s University, Tikur Anbessa Specialized Hospital, Nepalese Army Institute of Health Sciences, University of Cape Town, Kampala International University, Cleveland Clinic, University of Notre Dame d’Haiti, University of Birmingham, Obafemi Awolowo University, University of Ghana, Kaduna State University, School of Medicine and Health Sciences TecSalud, University of Malawi, University of Cincinnati, Kenyatta University, Royal College of Surgeons, Gazi University Tobruk University, Bond University, Jilin University, Christian Medical College & Hospital Ludhiana, Lund University, University of North Dakota, University of Zimbabwe, Hayat Medical College

C Percentage of years training could be 100% if a participant was in their intern year.

D To investigate the association between continuous variables and region, ANOVA was used. Chi-square or Fisher’s exact tests were used for categorical variables.

* Other medical degrees including but not limited to MB,BCh,BAO.

**Table 2:** Interests in medical student participation in research identified by survey participants

Topic	All participants (N=318)	Latin America (N=169)	North America (N=91)	Other (N=58)	OR (95% CI) of Latin America vs North America	OR (95% CI) of Other vs North America	p-value^A^
Interested in conducting research as a medical student, n (%)	277 (87.1)	140 (82.8)	83 (91.2)	54 (93.1%	0.54 (0.21, 1.44)	1.82 (0.45, 7.29)	0.095
Interested in conducting research later in medical training, n (%)	225 (70.8)	120 (71.0)	53 (58.2)	52 (89.7)	2.68 (1.35, 5.31)	7.96 (2.85, 22.28)	<0.001
Interested in conducting research as a part of your career as a physician, n (%)	187 (58.8)	103 (60.9)	36 (39.6)	48 (82.8)	3.80 (1.96, 7.36)	9.87 (4.02, 24.22)	<0.001
Most students in your medical institution participate in research, n (%)	106 (33.9)	35 (20.7)	61 (70.1)	10 (17.5)	0.10 (0.05, 0.20)	0.10 (0.04, 0.24)	<0.001
Research is an important component of training, n (%)	277 (87.7)	156 (92.3)	67 (75.3)	54 (93.1)	4.26 (1.82, 9.96)	4.93 (1.49, 16.32)	0.001
Important for physicians to participate in research, n (%)	288 (90.9)	160 (94.7)	71 (78.9)	57 (98.3)	-	-	<0.001
Motivating factors of research: Interest in being a physician scientist, n (%)	62 (20.0)	39 (23.8)	3 (3.4)	20 (35.1)	-	-	<0.001
Motivating factors of research: Competitiveness for matching to specialty training after medical school, n (%)	194 (62.6)	95 (57.9)	69 (77.5)	30 (52.6)	0.44 (0.23, 0.83)	0.37 (0.17, 0.79)	0.027

A Logistic regression models were used to investigate the effect of region on interests in medical school participation in research, adjusting for age, gender, and percentage of years training completed. Adjusted odds ratios (OR) and 95% confidence intervals (CI) were obtained. ‘Interested in conducting research later in medical training’ and ‘interested in conducting research as a part of your career as a physician’ were modeled ‘yes’ vs ‘maybe/no’. Fisher’s exact test was used for ‘Important for physicians to participate in research’ and ‘Motivating factors of research: Interest in being a physician scientist’ due to cell counts.

**Table 3:** Barriers to medical student participation in research identified by survey participants

Topic	All participants (N=318)	Latin America (N=169)	North America (N=91)	Other (N=58)	OR (95% CI) of Latin America vs North America	OR (95% CI) of Other vs North America	p-value^A^
Lack of research mentors, n (%)	172 (56.6)	91 (55.8)	42 (48.8)	39 (70.9)	1.82 (0.97, 3.42)	4.06 (1.76, 9.34)	0.005
Lack of available research project opportunities, n (%)	211 (69.4)	112 (68.7)	53 (61.6)	46 (83.6)	1.68 (0.86, 3.25)	3.62 (1.40, 9.32)	0.028
Lack of physical resources (computer or internet access), n (%)	16 (5.3)	7 (4.3)	2 (2.3)	7 (12.7)	-	-	0.029
Lack of research resources (database or statistical software), n (%)	55 (18.1)	24 (14.7)	8 (9.3)	23 (41.8)	1.44 (0.57, 3.64)	5.61 (2.11, 14.92)	<0.001
Lack of financial resources for publication/presentation, n (%)	124 (40.8)	77 (47.2)	13 (15.1)	34 (61.8)	5.62 (2.72, 11.60)	10.52 (4.42, 25.04)	<0.001
Lack of access to medical research articles, n (%)	36 (11.8)	15 (9.2)	3 (3.5)	18 (32.7)	2.60 (0.69, 9.81)	13.66 (3.55, 52.58)	<0.001
Lack of ability to attend conferences, n (%)	87 (28.6)	57 (35.0)	8 (9.3)	22 (40.0)	5.55 (2.39, 12.89)	7.79 (2.99, 20.33)	<0.001
Lack of ‘culture research’ at your institution, n (%)	115 (37.8)	71 (43.6)	12 (14.0)	32 (58.2)	6.44 (2.99, 13.88)	12.88 (5.19, 31.97)	<0.001
COVID19 Pandemic, n (%)	150 (49.3)	79 (48.5)	53 (61.6)	18 (32.7)	0.68 (0.38, 1.25)	0.43 (0.20, 0.93)	0.097
Lack of time for research, n (%)	4 (1.3)	2 (1.2)	2 (2.3)	0 (0.0)	-	-	0.646
Willing to participate in research if barriers were removed, n (%)	294 (94.5)	158 (94.6)	83 (92.2)	53 (98.1)	-	-	0.342

A Logistic regression models were used to investigate the effect of region on barriers to medical student participation in research, adjusting for age, gender, and percentage of years training completed. Adjusted odds ratios (OR) and 95% confidence intervals (CI) were obtained. Fisher’s exact tests were used for ‘Lack of physical resources’, ‘Lack of time for research’, and ‘Willing to participate in research if barriers were removed’ due to model fit.

## Key Messages

- Medical students across the globe unanimously agree that research is an important component of their medical training.
- Many medical students report a lack of research support from their institutions.
- Reported barriers to research included lack of research opportunities, mentorship and formal incorporation of research education in their training, in addition to funding to travel to conferences to present their work.
- Medical students from outside of the North American region reported interest in pursuing research later in their medical career at higher percentages than those in the North American region.

## References

[1] STARSurg Collaborative. Medical research and audit skills training for undergraduates: an international analysis and student-focused needs assessment. Postgrad Med J. 2018;94: 37–42. 28866608 10.1136/postgradmedj-2017-135035

[2] Amgad M, Man Kin Tsui M, Liptrott SJ, Shash E. Medical student research: an integrated mixed-methods systematic review and meta-analysis. PLoS One. 2015;10: e0127470. 26086391 10.1371/journal.pone.0127470 PMC4472353

[3] Metcalfe D Involving medical students in research. J R Soc Med. 2008;101: 102–103. 18344462 10.1258/jrsm.2008.070393 PMC2270240

[4] Cursiefen C, Altunbas A. Contribution of medical student research to the Medline-indexed publications of a German medical faculty. Med Educ. 1998;32: 439–440. 9743810 10.1046/j.1365-2923.1998.00255.x

[5] Aslam F, Shakir M, Qayyum MA. Why medical students are crucial to the future of research in South Asia. PLoS Med. 2005;2: e322. 16288553 10.1371/journal.pmed.0020322 PMC1297528

[6] Sorial AK, Harrison-Holland M, Young HS. The impact of research intercalation during medical school on postgraduate career progression. BMC Med Educ. 2021;21: 39. 33419435 10.1186/s12909-020-02478-7 PMC7792318

[7] Al-Busaidi IS, Wells CI, Wilkinson TJ. Publication in a medical student journal predicts short- and long-term academic success: a matched-cohort study. BMC Med Educ. 2019;19: 271. 31324236 10.1186/s12909-019-1704-x PMC6642564

[8] Möller R, Shoshan M. Medical students’ research productivity and career preferences; a 2-year prospective follow-up study. BMC Med Educ. 2017;17: 51. 28253880 10.1186/s12909-017-0890-7 PMC5335804

[9] Arshad S, McCombe G, Carberry C, Harrold A, Cullen W. What factors influence medical students to enter a career in general practice? A scoping review. Ir J Med Sci. 2021;190: 657–665. 32794065 10.1007/s11845-020-02345-w

[10] Jones M, Hutt P, Eastwood S, Singh S. Impact of an intercalated BSc on medical student performance and careers: a BEME systematic review: BEME Guide No. 28. Med Teach. 2013;35: e1493–510. 23962229 10.3109/0142159X.2013.806983

[11] Nazha B, Salloum RH, Fahed AC, Nabulsi M. Students’ perceptions of peer-organized extra-curricular research course during medical school: a qualitative study. PLoS One. 2015;10: e0119375. 25764441 10.1371/journal.pone.0119375 PMC4357456

[12] Carberry C, McCombe G, Tobin H, Curriculum initiatives to enhance research skills acquisition by medical students: a scoping review. BMC Med Educ. 2021;21: 312. 34078364 10.1186/s12909-021-02754-0 PMC8173745

[13] Salloum RH, Nazha B, Zgheib NK. Interest and Involvement in Research During Medical School: A Global Comparison of Students at High- and Low-Income Universities. Medical Science Educator. 2014;24: 65–73.

[14] Reinders JJ, Kropmans TJB, Cohen-Schotanus J. Extracurricular research experience of medical students and their scientific output after graduation. Med Educ. 2005;39: 237. 15679693 10.1111/j.1365-2929.2004.02078.x

[15] Jacobs CD, Cross PC. The value of medical student research: the experience at Stanford University School of Medicine. Med Educ. 1995;29: 342–346. 8699971 10.1111/j.1365-2923.1995.tb00023.x

[16] Negida A Egypt’s premier medical student research group: a new model for medical student research in developing countries. Cureus. 2018. doi:10.7759/cureus.3561 PMC6325031 30648093

[17] Fallah PN, Jayaram A, Hauser BM. Moving the needle on global surgery education in the US. J Surg Educ. 2021;78: 1780–1782. 33965360 10.1016/j.jsurg.2021.04.006 PMC8883341

[18] Murdoch-Eaton D, Drewery S, Elton S, What do medical students understand by research and research skills? Identifying research opportunities within undergraduate projects. Med Teach. 2010;32: e152–60. 20218832 10.3109/01421591003657493

[19] Weaver AN, McCaw TR, Fifolt M, Hites L, Lorenz RG. Impact of elective versus required medical school research experiences on career outcomes. J Investig Med. 2017;65: 942–948. 10.1136/jim-2016-000352 PMC5833985 28270407

[20] Diez C, Arkenau C, Meyer-Wentrup F. The German medical dissertation--time to change? Acad Med. 2000;75: 861–863. 10965870 10.1097/00001888-200008000-00024

